# Mediating Effect of Sleep Quality on the Relationship Between Problematic Mobile Phone Use and Depressive Symptoms in College Students

**DOI:** 10.3389/fpsyt.2019.00822

**Published:** 2019-11-13

**Authors:** Liwei Zou, Xiaoyan Wu, Shuman Tao, Honglv Xu, Yang Xie, Yajuan Yang, Fangbiao Tao

**Affiliations:** ^1^Department of Maternal, Child and Adolescent Health, School of Public Health, Anhui Medical University, Hefei, China; ^2^Anhui Provincial Key Laboratory of Population Health and Aristogenics, Anhui Medical University, Hefei, China; ^3^MOE Key Laboratory of Population Health Across Life Cycle, Hefei, China; ^4^Department of Nephrology, the Second Hospital of Anhui Medical University, Hefei, China; ^5^School of Nursing, Anhui Medical University, Hefei, China

**Keywords:** problematic mobile phone use, sleep quality, depression, mediation, adolescents

## Abstract

**Background and Aim:** Problematic mobile phone use (PMPU) and depression are great public health concerns among adolescents. The aim of this study was to determine the association between PMPU and symptoms of depression, as well as the mediating role of sleep quality.

**Methods:** A total of 4,624 college students participated in this study. The Self-rating Questionnaire for Adolescent Problematic Mobile Phone Use (SQAPMPU), Pittsburgh Sleep Quality Index (PSQI), and Patient Health Questionnaire-9 (PHQ-9) were administered to assess PMPU, sleep quality, and symptoms of depression. Mediation analysis was conducted using PROCESS macro in the SPSS software.

**Results:** Of the participants, 27.5% were classified as PMPU, 44.9% exhibited symptoms of depression, and 15.6% reported sleep problems. Compared with those without PMPU, those with PMPU exhibited higher rates of sleep problems and depressive symptoms. The mediation analysis further revealed partial mediation effects of sleep quality on the association between PMPU and depression.

**Conclusions:** This study demonstrated that PMPU was associated with mental health in college students, and sleep quality played a mediating role in this relationship. Our findings highlight the critical role of early intervention for depression with a focus on those with PMPU and, more specifically, on those with sleep problems.

## Introduction

Depression is a common mental health problem among college students, with a prevalence ranging from 1.5% to 19% (1, 2). It has been reported that depression is the second leading cause of disability-adjusted life years (DALYs) which are characterized by physical, emotional, and/or psychosocial dysfunction (3–5). For example, depression can cause poor academic performance, cognitive impairment, and sleep problems (6–8). Research has found that depression among adolescents is associated with certain behaviors, such as substance use, earlier sexual experiences, problem internet use, problem mobile phone use (PMPU), and so on (9–12). Hence, there has been a considerable increase in research focusing on the relationship of depression, sleep quality, and PMPU.

Mobile phones have become increasingly more necessary electronic media devices in daily life, especially given their ability to access the internet (13). Mobile phone use is also a considerable concern, especially mobile phone use among adolescents. It has been reported that 6.3% of high school students in Italy (14), 16% of middle school students in Korea (15), 20% of high school students in Spain (16), and 26% of the young Tunisian population (17) exhibit signs of PMPU. PMPU is becoming more widespread and leads to physical, psychological, and social impairments (18–21). Several studies have found that PMPU is correlated with depression. There are also significant correlations between PMPU and age, impulsiveness, and excessive reassurance seeking (22). Visnjic et al. suggested that the intensity and modality of mobile phone use could affect the mental health of university students (12). A longitudinal study found that greater mobile phone use at baseline was associated with higher depression scores at a 1-year follow up, a finding that could provide new insight for the relationship between mobile phone use and the development of depression symptoms (23).

Meanwhile, sleep quality is well known to be associated with depression among young adults. Previous research has suggested sleeping less than 6 h per day leads to an increased incidence of depression among adolescents (24). Another study found that short sleep onset latency and a refusal to sleep are significant independent predictors of major depressive disorder (25). Furthermore, several researchers have identified inverse associations between sleep quality and depression. A meta-analysis found that adolescents with major depressive disorder have poor sleep quality, including more sleep disruptions, sleep disturbances, insomnia, and hypersomnia (26). Adolescents with depression reported significantly longer sleep onset latencies, more sleep disruptions, and lower sleep efficiency than did the controls.

We hypothesized that PMPU is associated with sleep quality and symptoms of depression among young adults. The present study aimed to determine the association between PMPU and depression among college students. We also examined the mediating role of sleep quality on the relationship between PMPU and symptoms of depression.

## Methods

### Participants

This cross-sectional study was performed between May 2018 and June 2018 to assess the behaviors and mental health of college students. Participants were selected from two universities, one located in Anhui province and the other in Jiangxi province. In collaboration with the two universities, we visited each university and described the study and the questionnaires to the students. A QR code for scanning using their cell phones were given to the students, thus allowing them to access the electronic questionnaires. A total of 4,624 university students (2,058 males and 2,566 females) aged between 17 and 25 years old (mean ± SD: 19.91 ± 1.27 years) participated in the study. The response rate was 96.59%. This study was reviewed and approved by the ethics committee of Anhui Medical University. Written informed consent was obtained from all participants prior to the administration of the survey.

### Measures

The information collected included sociodemographic data, such as age, gender, and residential area (rural/urban), as well as three measures regarding specific symptomatic conditions, namely, the Self-rating Questionnaire for Adolescent Problematic Mobile Phone Use (SQAPMPU), the Patient Health Questionnaire-9 (PHQ-9), and the Pittsburgh Sleep Quality Index (PSQI).

Self-rating Questionnaire for Adolescent Problematic Mobile Phone Use (SQAPMPU) (27): The SQAPMPU is used to assess PMPU among college students. The questionnaire consists of 13-items (e.g., “I always feel I do not have enough time to use my mobile phone,” “My leisure activities are reduced due to the time I spend on my mobile phone,” “I become irritable if I have to switch off my mobile phone for meetings, dinner engagements, or at the movies”). Responses were given on a 5-point Likert scale (not true at all = 1, slightly true = 2, moderately true = 3, strongly true = 4, extremely true = 5). This questionnaire assessed three dimensions including withdrawal symptoms, craving, and physical and mental health statuses. The total scores ranged from 13 to 65, where participants with SQAPMPU scores ≥ 29 were defined as the PMPU group and those with SQAPMPU scores <29 were defined as the non-PMPU group. The 75^th^ percentile served as the cutoff point, and the Cronbach’s alpha coefficient was 0.93.

Patient Health Questionnaire-9 (PHQ-9) (28): The PHQ-9 is a nine-item self-report questionnaire that assesses depressive symptoms experienced during the last 2 weeks. Example items included “little interest or pleasure in doing things,” “feeling tired or having little energy” and “feeling down, depressed, or hopeless.” Each item on the measure contains four response options to describe how often the respondent experienced each feeling in the last 2 weeks: responses of 0, 1, 2, and 3 corresponded to “not at all,” “several days,” “more than half the days,” and “nearly every day,” respectively. The total scores ranged from 0 to 27, with higher scores indicating more severe depressive symptoms. The Cronbach’s alpha coefficient was 0.93.

Pittsburgh Sleep Quality Index (PSQI) (29): The PSQI is a self-reported questionnaire that assesses sleep quality over the last month. This measure consists of 19 items (e.g., “During the past month, how would you rate your sleep quality overall?”, “During the past month, how long has it usually taken you to fall asleep each night?”, “During the past month, how many hours of actual sleep did you get at night?”). This index assesses seven components of sleep, namely, sleep quality, sleep latency, sleep duration, sleep efficiency, sleep disturbance, use of sleeping medication, and daytime dysfunction. Sleep quality refers to the subject’s perceived overall sleep quality. Sleep latency refers to how long it takes to fall asleep. Sleep duration refers to the subject’s actual length of sleep. Sleep efficiency refers to how much time was set aside for sleeping and spent in bed. Sleep disturbance refers to behaviors that affect sleep at night, such as uncomfortable breathing, getting up at night to use the restroom, and so on. Each component is scored on a 0 (no difficulty) to 3 (severe difficulty) interval scale. The total scores are calculated by totaling the seven component scores; thus, total scores range from 0 to 21, where higher scores indicate poorer sleep quality.

### Statistical Analysis

All statistical analyses were performed using SPSS version 23.0 (SPSS, Chicago, IL, USA). For the descriptive analyses, means (SD) and median were used for continuous variables, and frequencies and percentages were used for categorical variables. T-test and chi-square analyses were performed to compare the demographic characteristics between the PMPU and non-PMPU groups. Multivariate logistic regression was performed to determine the association between the predictive variables and depressive symptoms. Pearson’s correlation analysis was conducted to determine the relationships between sleep quality and depression in the PMPU and non-PMPU groups. To examine whether sleep quality mediated the relationship between PMPU and depression, we performed a mediation analysis using the SPSS PROCESS macro, version 3.0 (model 4), developed by Hayes (30). Statistical significance was set at *p* < 0.05.

## Results

Participants with SQAPMPU scores ≥29 were defined as the PMPU group, while those with SQAPMPU scores <29 were defined as the non-PMPU group; the 75^th^ percentile was used as the cutoff point. Higher scores on the PHQ-9 indicate more severe depressive symptoms: none = 0–4, mild = 5–9, moderate = 10–14, moderately severe = 15–19, and severe = 20–27. For PSQI, we used a score of 7 as the cutoff point; thus, a total score ≤7 was classified as good sleep, and scores >7 were classified as poor sleep. We included 4,624 participants, with a mean age (SD) of 19.91 (1.27) years. Slightly more than half (52.1%) of the participants came from rural environments, and more than half (55.5%) of the participants were female. A total of 27.5% (n = 1,271) of the participants were in the PMPU group, while 72.5% (n = 3,353) were in the non-PMPU group. Moreover, 44.9% (n = 2,546) of the total sample experienced probable depression (PHQ-9 ≥ 5), and 15.6% (n = 720) of the participants reported sleep problems (PSQI ≥ 8).

**Table 1** shows the results when comparing age, gender, residential area, PSQI scores, and PHQ-9 scores between the PMPU group and the non-PMPU group. Compared with non-PMPU group, the PMPU group exhibited higher PSQI and PHQ-9 scores (Cohen’s d = 0.819, *p* < 0.001, and Cohen’s d = 1.164, *p* < 0.001, respectively). Those with PMPU were more likely to have sleep problems and experience depression. No age, gender, or residential area differences were found between the two groups.

**Table 1:** Demographic characteristics of adolescents with and without problem mobile phone use.

	Total (n = 4,624)	PMPU group (n = 1,271)	Non-PMPU group (n = 3,353)	Test	Cohen’s d	*P* value
Age (years), mean±SD	19.91±1.27	19.94±1.24	19.90±1.28	*t* = −1.18	0.039	0.239
Gender						
Male (n, %)	2,058 (44.5%)	570 (44.8%)	1,488 (44.4%)	χ*^2^* = 0.08	0.004	0.775
Female (n, %)	2,566 (55.5%)	701 (55.2%)	1,865 (55.6%)			
Residential area						
Rural (n, %)	2,411 (52.1%)	658 (51.8%)	1,753 (52.3%)	χ*^2^* = 0.10	0.005	0.756
Urban (n, %)	2,213 (47.9%)	613 (48.2%)	1,600 (47.7%)			
Sleep quality (PSQI global score: mean±SD)	4.84±0.42	6.42±3.09	4.24±2.48	*t* = −24.85	0.819	<0.001
Subjective sleep quality						
0 (n, %)	940 (20.3%)	187 (14.7%)	753 (22.5%)	χ*^2^* = 241.49	15.540	<0.001
1 (n, %)	2,718 (58.8%)	631 (49.6%)	2,087 (62.2%)			
2 (n, %)	819 (17.7%)	371 (29.2%)	448 (13.4%)			
3 (n, %)	147(3.2%)	82 (6.5%)	65 (1.9%)			
Sleep latency						
0 (n, %)	1,552 (33.6%)	256 (20.1%)	1,296 (38.7%)	χ*^2^* = 276.50	16.628	<0.001
1 (n, %)	1,864 (40.3%)	478 (37.6%)	1,386 (41.3%)			
2 (n, %)	871 (18.8%)	372 (29.3%)	499 (14.9%)			
3 (n, %)	337 (7.3%)	165 (13.0%)	172 (5.1%)			
Sleep duration						
0 (n, %)	1,940 (42.0%)	461 (36.3%)	1,479 (44.1%)	χ*^2^* = 57.55	7.586	<0.001
1 (n, %)	1,726 (37.3%)	456 (35.9%)	1,270 (37.9%)			
2 (n, %)	958 (20.7%)	354 (27.9%)	604 (18.0%)			
3 (n, %)	0	0 (0%)	0 (0%)			
Habitual sleep efficiency						
0 (n, %)	3,496 (75.6%)	885 (69.6%)	2,611 (77.9%)	χ*^2^* = 38.02	6.166	<0.001
1 (n, %)	588 (12.7%)	187 (14.7%)	401 (12.0%)			
2 (n, %)	221(4.8%)	85 (6.7%)	136 (4.1%)			
3 (n, %)	319 (6.9%)	114 (9.0%)	205 (6.1%)			
Sleep disturbance						
0 (n, %)	1,066 (23.1%)	107 (8.4%)	959 (28.6%)	χ*^2^* = 601.61	24.528	<0.001
1 (n, %)	2,985 (64.6%)	786 (61.8%)	2,199 (65.6%)			
2 (n, %)	500 (10.8%)	318 (25.0%)	182 (5.4%)			
3 (n, %)	73 (1.6%)	60 (4.7%)	13 (0.4%)			
Use of sleeping medications						
0 (n, %)	4,338 (93.8%)	1,115 (87.7%)	3,223 (96.1%)	χ*^2^* = 116.99	10.816	<0.001
1 (n, %)	168 (3.6%)	90 (7.1%)	78 (2.3%)			
2 (n, %)	73 (1.6%)	46 (3.6%)	27 (0.8%)			
3 (n, %)	45 (1.0%)	20 (1.6%)	25 (0.7%)			
Daytime dysfunction						
0 (n, %)	2,326 (50.3%)	386 (30.4%)	1,940 (57.9%)	χ*^2^* = 368.60	19.199	<0.001
1 (n, %)	1,989 (43.0%)	699 (55.0%)	1,290 (38.5%)			
2 (n, %)	253 (5.5%)	145 (11.4%)	108 (3.2%)			
3 (n, %)	56 (1.2%)	41 (3.2%)	15 (0.4%)			
Depression symptom (PHQ global score: mean±SD)	4.89±0.08	8.87±6.18	3.39±4.01	*t* = −35.35	1.164	<0.001

PSQI, Pittsburgh Sleep Quality Index; PHQ-9, Patient Health Questionnaire-9, PMPU, problematic mobile phone use.

**Table 2** shows the results of the multiple logistic regression analysis. The results show that sleep problems (OR = 1.48, 95% CI = 1.43∼1.53) and PMPU (OR = 1.11, 95% CI = 1.10∼1.12) increase the risk of depressive symptoms. Moreover, sleep problems, as measured by the PSQI, were significantly correlated with depression in both the PMPU and non-PMPU groups (*r* = 0.52, *p* < 0.001, and *r* = 0.48, *p* < 0.001, respectively) (**Table 3**).

**Table 2:** Results of logistic regression.

	Depressive symptom		
	OR	95% *CI*	*P* value
SQAPMPU score	1.11	1.10–1.12	<0.001
PSQI global score	1.48	1.43–1.53	<0.001

OR, odds ratio; CI, confidence interval; SQAPMPU, Self-rating Questionnaire for Adolescent Problematic Mobile Phone Use scale; PSQI, Pittsburgh Sleep Quality Index. Adjusted factors include age, gender, and residential area.

**Table 3:** Correlation of the variables in PMPU and non-PMPU group.

PMPU group (n = 1,271)	Depressive
Sleep quality	0.52**
Depressive	–
Non-PMPU group (n = 3,353)	Depressive
Sleep quality	0.48**
Depressive	–

**p < 0.001.

**Figure 1:**
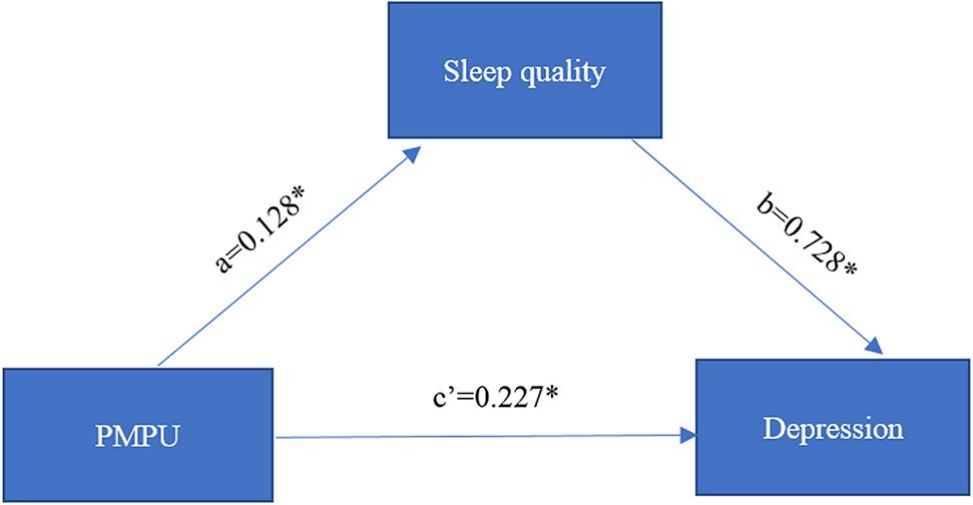
The estimated coefficients of mediation effects of sleep quality on problematic mobile phone use (PMPU) and depression. * p < 0.05.

The model of the relationship between PMPU and depression mediated by sleep quality is shown in **Figure 1**. Because gender was correlated with sleep problems and depression in the non-PMPU group, we controlled for gender as a covariate in this model. The mediation model was significant (*F* = 1,195.70, *p* < 0.001), accounting for 29% (0.093/0.227+0.093) of the mediating effect, where the total effect is equal to the direct effect (0.027) plus the indirect effect (0.093). Both the direct effect of PMPU on sleep quality (*a* = 0.128, SE = 0.004, *p* < 0.001) and the quality of sleep on depression (*b* = 0.728, SE = 0.022, *p* < 0.001) were significant. Moreover, the direct effect of PMPU on depression (*c’* = 0.227, SE = 0.006, *p* < 0.001) was also significant. Finally, the bootstrapping results proved the significant effect of PMPU on depression through sleep problems [*B* = 0.093, bias-corrected and accelerated 5,000 bootstrapping (BCa) 95% CI = 0.083∼0.103] (**Table 4**). The full model explains 35% of the total variance of PMPU on depression. These results suggest that sleep quality partially mediates the relationship between PMPU and depression.

**Table 4:** Summary of mediation analysis of sleep quality between PMPU and depressive.

Paths	B	SE	*t*	BCa 95% CI	
				Lower	Upper
PMPU → sleep quality	0.128	0.004	32.902**		
Sleep quality → depressive	0.728	0.022	32.542**		
PMPU → depressive	0.227	0.006	34.625**		
PMPU → sleep quality → depressive	0.093	0.005	–	0.083	0.103

**p < 0.001. BCa, bias-corrected and accelerated 5,000 bootstrapping.

## Discussion

To the best of our knowledge, this is the first large-scale study that aimed to investigate the association between PMPU and symptoms of depression as well as sleep quality in college students and to estimate the mediating effect of sleep quality. The results of this study indicate that more than one-fourth of the sample (27.5%) exhibited PMPU. Students with PMPU experience more sleep problems and exhibit more symptoms of depression compared with non-PMPU students. The study also demonstrates that PMPU contributes to depression due to its negative impact on sleep quality.

Regarding the pathway from PMPU to sleep quality, our results are consistent with previous studies that find a significant association between PMPU and sleep quality (31, 32). This association may be directly related to the fact that college students use their mobile phones while in bed before falling asleep. One explanation for this association suggests that college students want to prolong their time online, which results in a sleep phase shift to later hours. In addition, excessive mobile phone use leads to sleep disturbances due to irregular bedtime patterns (33, 34).

There is also a significant association between sleep quality and symptoms of depression, which is consistent with previous results (35, 36). However, we did not find any gender-based difference between the PMPU and non-PMPU groups, whereas Liu et al. reported that the prevalence of PMPU was higher among females than among males (31). This difference may be because females use mobile phones more often to access social networking sites such as Facebook and use them more frequently for bloggings, online shopping, text messaging, and watching videos. Another interesting study (37) revealed that male Japanese problematic internet users have an interpersonal fear tendency and avoid social interaction.

For psychiatric symptoms, our results were not much different from those of other studies. However, we did find that sleep quality mediates the effect of PMPU on symptoms of depression. Adams et al. (38) suggested that sleep quality acts as a mediator between technology use after sleep onset and depression and anxiety. Similarly, Xie et al. (39) found that problematic smartphone use was harmful to both mental and physical health and emphasized the importance of sleep quality in mediating the effect of problematic smartphone use on health symptoms among adolescents. Furthermore, a study on problematic internet use revealed the mediating effect of insomnia on the association between problematic internet use and depression among secondary school students (40). Although it was a relatively small sample, this finding demonstrates the partially mediating effect of sleep problems on the association between problematic media device use and mental health, consistent with our findings, which supports the “displacement hypothesis” theory (41).

Two mechanisms related to neurobiological pathways may explain the mediating effect of sleep quality on the association between PMPU and symptoms of depression. First, the role of sleep quality on depression may be related to circadian rhythms. It has been reported that the circadian rhythm plays a key role in affective disorders, such as bipolar disorder and depression (42, 43). During adolescence, the evening chronotype experiences a dramatic change, which acts as a vulnerability marker of emotional difficulties (13). Relatedly, the blue light exposure from a mobile phone may suppress the secretion of melatonin, which in turn delays the circadian rhythm (11). Second, the hypothalamic–pituitary–adrenal (HPA) axis is regarded as a physiological link between affective disorders and sleep quality. Although the results from depressive adolescents are less consistent, they suggest that the HPA axis is correlated with depression (18, 19, 44). Hence, sleep quality may influence HPA functions and thus lead to increases in depression. A plausible explanation for the mediation effects of sleep quality may be that PMPU can disrupt college students’ normal sleep patterns, which then contributes to a high risk of depression.

The present study, which focused on a public problem and highlights the significance of the prevention of PMPU among college students, found that to reduce the negative effects of PMPU, it is necessary not only to control the time of mobile phone use but also to ensure sufficient sleep time and sleep quality. Even though the underlying mechanism of depression remains unclear, it may arise from the interactions of many factors, such as PMPU and sleep problems.

Our study has several limitations. First, although our data were collected from well-validated self-reported questionnaires, the study cannot mask the *ad hoc* nature of the measures used herein and the effects of recall bias. Second, although this is a large-scale, cross-sectional design study, it cannot detect a causal relationship, although we assume the direction is behavior to emotional symptoms. A future longitudinal study is suggested. Third, we failed to directly test the time and content of mobile phone use as PMPU was measured by the SQAPMPU, which was not conducted in this study due to a limitation of resources. Finally, although sleep quality may mediate the association between PMPU and other mental health concerns, the current study only considered the symptoms of depression. Our future studies will consider problems such as anxiety and stress. Despite these limitations, our study extends the previous results regarding the association between PMPU, sleep problems, and depression by estimating the mediating effects of sleep quality on the association between PMPU and depression among college students.

In summary, our study detected a mediating effect of sleep quality on the relationship between PMPU and depression. These results suggest the importance of early intervention among adolescents with PMPU, especially among those with sleep problems.

## Data Availability Statement

The datasets generated for this study are available on request to the corresponding author.

## Ethics Statement

The studies involving human participants were reviewed and approved by the Ethics Committee of Anhui Medical University. The patients/participants provided their written informed consent to participate in this study.

## Author Contributions

FT design the study. LZ, XW, ST, HX, YX, and YY performed the survey research. LZ, XW, ST, and HX analyzed the data. LZ draft the manuscript. Finally, all authors read and approval the final manuscript.

## Funding

This study was supported by National Natural Science Foundation of China (81773455, 81803257), Scientific Research of BSKY from Anhui Medical University (XJ201824).

## Conflict of Interest

The authors declare that the research was conducted in the absence of any commercial or financial relationships that could be construed as a potential conflict of interest.
